# Targeted CRM1-inhibition perturbs leukemogenic NUP214 fusion proteins and exerts anti-cancer effects in leukemia cell lines with *NUP214* rearrangements

**DOI:** 10.18632/oncotarget.27711

**Published:** 2020-09-08

**Authors:** Adélia Mendes, Ramona Jühlen, Valérie Martinelli, Birthe Fahrenkrog

**Affiliations:** ^1^Institute of Molecular Biology and Medicine, Université Libre de Bruxelles, Charleroi 6041, Belgium; ^2^Institute of Biochemistry and Molecular Cell Biology, RWTH Aachen University, Aachen 52074, Germany

**Keywords:** CRM1, NUP214, leukemia, nuclear export, nucleoporin

## Abstract

Chromosomal translocations fusing the locus of nucleoporin *NUP214* each with the proto-oncogenes *SET* and *DEK* are recurrent in, largely intractable, acute leukemias. The molecular basis underlying the pathogenesis of SET-NUP214 and DEK-NUP214 are still poorly understood, but both chimeras inhibit protein nuclear export mediated by the β-karyopherin CRM1. In this report, we show that SET-NUP214 and DEK-NUP214 both disturb the localization of proteins essential for nucleocytoplasmic transport, in particular for CRM1-mediated protein export. Endogenous and exogenous SET-NUP214 and DEK-NUP214 form nuclear bodies. These nuclear bodies disperse upon targeted inhibition of CRM1 and the two fusion proteins re-localize throughout the nucleoplasm. Moreover, SET-NUP214 and DEK-NUP214 nuclear bodies reestablish shortly after removal of CRM1 inhibitors. Likewise, cell viability, metabolism, and proliferation of leukemia cell lines harboring SET-NUP214 and DEK-NUP214 are compromised by CRM1 inhibition, which is even sustained after clearance from CRM1 antagonists. Our results indicate CRM1 as a possible therapeutic target in NUP214-related leukemia. This is especially important, since no specific or targeted treatment options for NUP214 driven leukemia are available yet.

## INTRODUCTION

**Figure 1:**
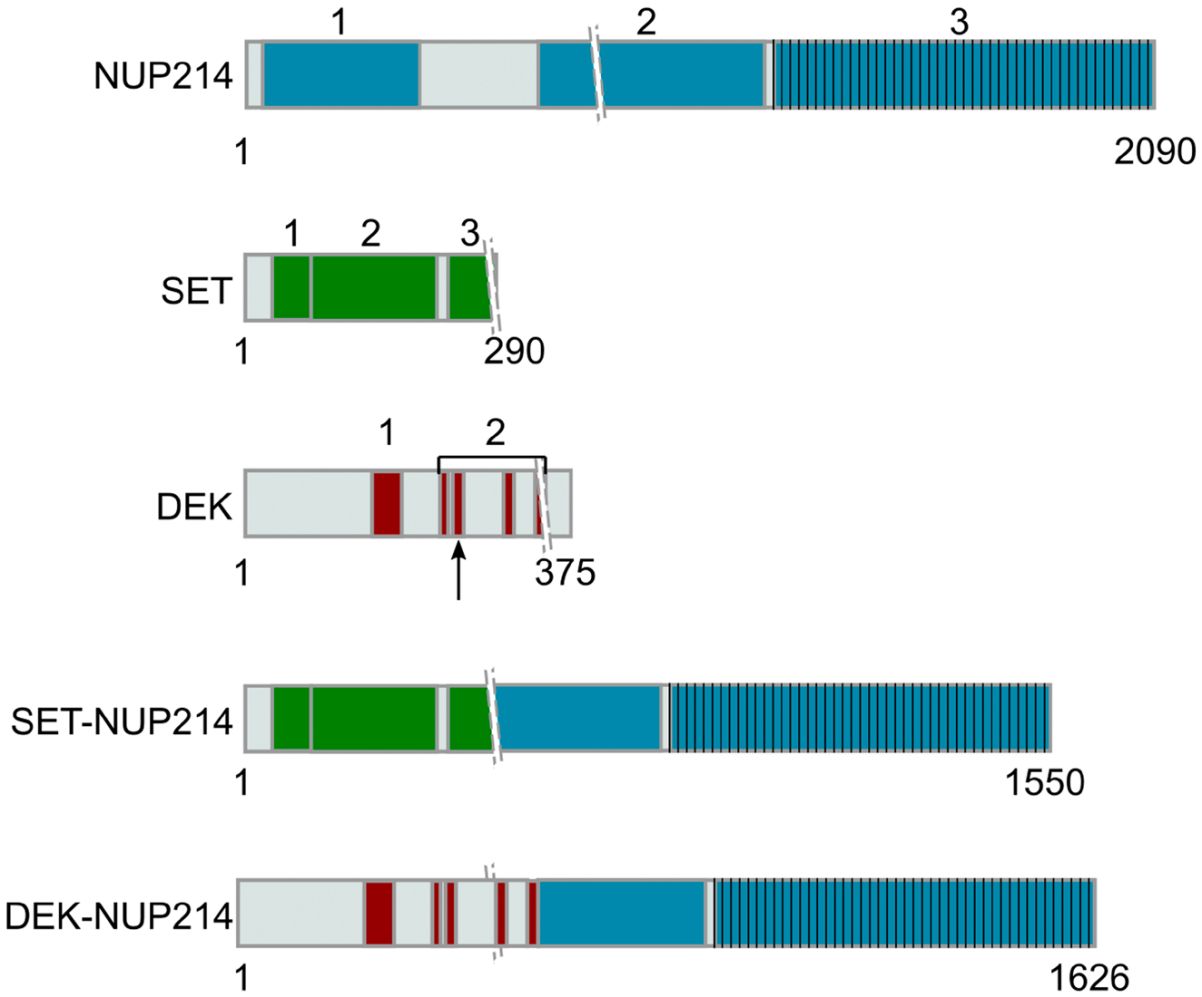
Representation of NUP214 and its binding partners in leukemogenic NUP214 fusion proteins. The numbers indicate the specific domains of each protein. Crossing lines (\\) represent the breakpoints in the respective fusion protein. NUP214: 1— β-propeller, 2— Coiled coil, 3— FG domain; SET: 1 ––dimerization domain, 2— earmuff domain, 3— acidic domain; DEK: 1— scaffold attachment factor (SAF)-box domain (DNA-binding domain), 2— acidic domains (overlaps with the second DNA binding domain, represented by the arrow).

Chromosomal translocations involving the nucleoporin *NUP214* have been described in *de novo* and therapy-related acute myeloid leukemia (AML) as well as acute lymphoblastic leukemia (ALL). NUP214-related malignancies are frequently associated with poor treatment response and poor prognosis [1–7]. The fusion proteins SET-NUP214 [del (9)(q34.11q34.13)] and DEK-NUP214 [t (6;9)(p23; q34)] result from the fusion of the almost entire SET and DEK proteins with the C-terminal part of NUP214 (Figure 1) [1, 8, 9]. NUP214 is an integral part of the nuclear pore complex (NPC) and it plays important roles in nuclear export mediated by chromosomal region maintenance 1 (CRM1, or exportin 1/XPO1) [10–13]. CRM1 is the major nuclear export receptor for proteins and ribonucleoprotein (RNP) complexes carrying a characteristic nuclear export signal (NES) [14–17]. NUP214 functions as a terminal docking site for CRM1 nuclear export complexes on the cytoplasmic side of NPCs and depletion of NUP214 results in nuclear accumulation of NES-containing cargoes [18–21].

The C-terminal phenylalanine-glycine (FG) repeat domain of NUP214 exhibits multiple CRM1-binding sites, which are preserved in SET-NUP214 and DEK-NUP214 [21–24]. In fact, both fusion proteins can bind CRM1 and its co-factor, the small GTPase Ran, and inhibit the nuclear export of NES-containing proteins and RNPs [22, 23, 25]. Targeted CRM1 inhibition by small molecule antagonists has become an appealing anti-cancer strategy, for both solid and hematologic malignancies [26–40]. Leptomycin B (LMB), a fungal metabolite from *Streptomyces* spp, was the first identified small molecule inhibitor specifically targeting CRM1 [41]. LMB has potent anti-cancer activity, but its application in patients was withdrawn after a single phase I clinical trial because of its low efficiency and high toxicity [42–44]. Selective inhibitors of nuclear export (SINEs) comprise a novel class of CRM1 antagonists with anti-cancer properties both *in vitro* and *in vivo* [26–29, 45–47]. Indeed, the SINE compound KPT-330 is currently tested in phase 2/3 clinical trials for a wide variety of cancers, including leukemia and other hematologic malignancies [48]. The anti-cancer effects of CRM1 inhibitors are based on the induction of cell death by apoptosis and on cell cycle arrest due to activation of the transcriptional programs of tumor suppressor genes, such as *TP53*, *RB1*, and *FOXO*-related tumor suppressors [26, 27, 32]. A recent study in the context of refractory metastatic prostate cancer moreover revealed a direct link between nuclear retention of the mRNA of the eIF4E translation factor and inhibition of tumor cell growth due to SINE-mediated export inhibition [49]. Moreover, SINEs were recently approved for the treatment of refractory multiple myeloma, in combination with anti-myeloma drugs. Besides the inhibition of nuclear export of tumor suppressors, SINEs treatment was shown to further reduce the export of mRNAs which rely on CRM1-bound eEIF4 for export [49]. Despite the functional proximity between NUP214 and CRM1-mediated nuclear export and the functional relevance of CRM1 in some leukemia, the impact of CRM1 inhibition in the context of NUP214-related leukemia has not been studied yet.

Here we used patient-derived leukemia cell lines expressing endogenous SET-NUP214 and DEK-NUP214 to address the anti-cancer potential of CRM1 inhibition in NUP214-rearranged leukemia. We report that CRM1 inhibition by LMB or the SINE compound KPT-185 is sufficient to disturb the localization of endogenous SET-NUP214 and DEK-NUP214, coinciding with reduced cell viability and proliferation. CRM1 inhibition reduces cell viability and metabolic activity in a sustained manner after drug withdrawal. However, after drug removal, proliferation and SET-NUP214 and DEK-NUP214 nuclear bodies are restored. Our data suggest that CRM1 constraint is an interesting candidate for the development of an anti-cancer therapeutic approach in NUP214-related leukemia, for which no efficient targeted therapy has been developed so far.

## RESULTS

### Leukemogenic *NUP214* fusion proteins locate to nuclear bodies in patient-derived cells

**Figure 2:**
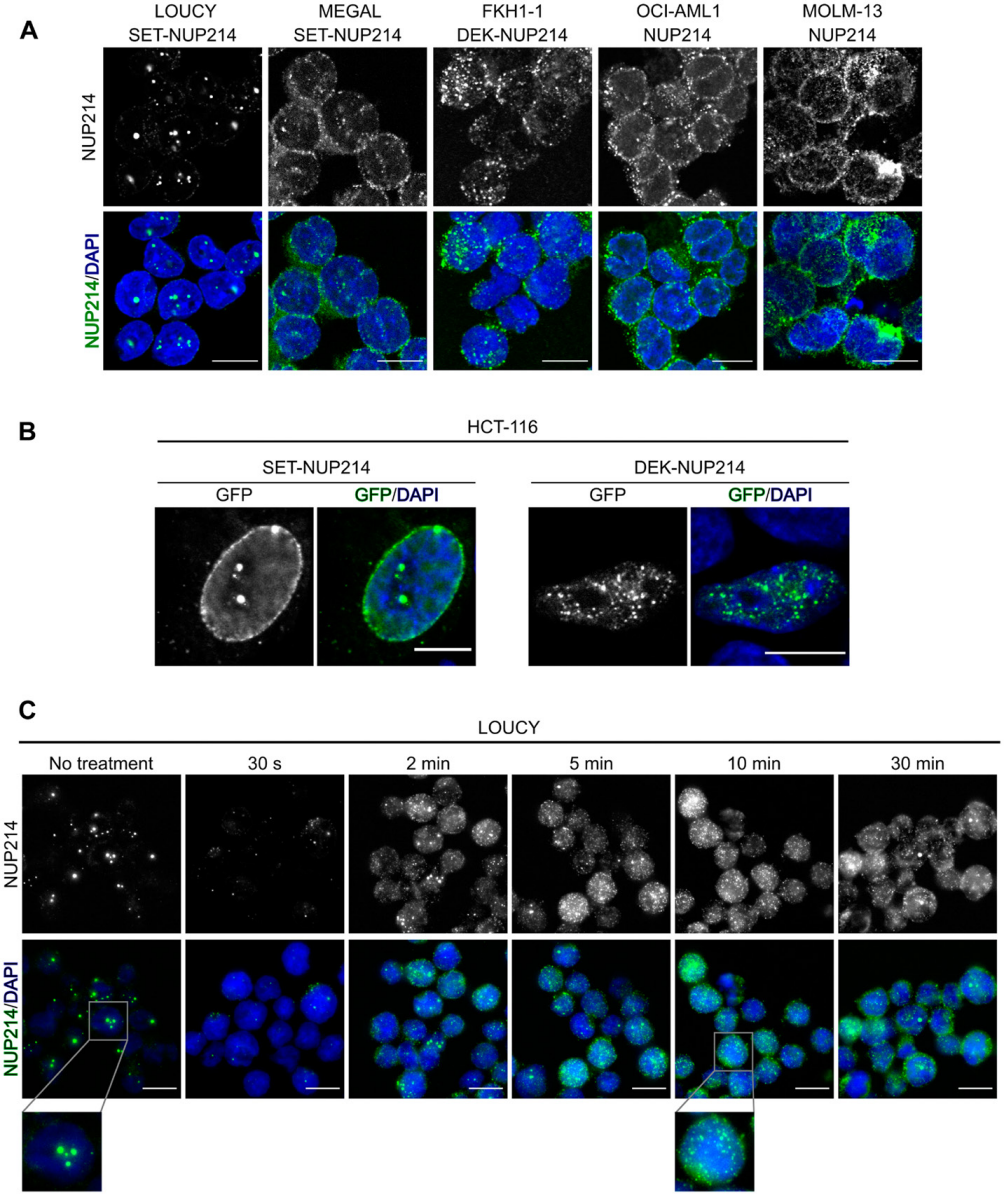
NUP214 fusion proteins localize to distinct nuclear bodies. (**A**) Cellular distribution of NUP214 in distinct leukemia cell lines. Foci correspond to nuclear bodies formed by SET-NUP214 (LOUCY and MEGAL) or DEK-NUP214 (FKH-1). (**B**) HCT-116 cells transfected with SET-NUP214-GFP or DEK-NUP214-GFP. (**C**) LOUCY cells were treated with 5% 1,6-hexanediol for the indicated time points. The presence of nuclear bodies was evaluated by immunofluorescence of NUP214 (green). DNA was visualized with DAPI (blue). Shown are representative epifluorescence (A, C) and confocal images (B). Scale bars, 10 μm.

We first determined the localization of NUP214 fusion proteins in different patient-derived leukemia cell lines with anti-NUP214 antibodies and immunofluorescence microscopy. LOUCY and MEGAL cells express SET-NUP214 (Figure 1) and in both cell lines SET-NUP214 located to the nuclear rim and to nuclear bodies (Figure 2A), consistent with previous results [22, 50]. FKH-1 cells harbor DEK-NUP214 (Figure 1), which localized to smaller nuclear bodies as compared to SET-NUP214 (Figure 2A) [51]. Similar localizations for GFP-tagged versions of SET-NUP214 and DEK-NUP214 were observed in transiently transfected HCT-116 cells (Figure 2B). In FKH-1 cells, NUP214 antibodies were also detected at the nuclear rim, which likely corresponds to endogenous NUP214 rather than to the fusion protein, as DEK-NUP214-GFP in HCT-116 was not detected at NPCs (Figure 2A and 2B). In OCI-AML1 and MOLM-13 cells, which do not express NUP214 fusion proteins, NUP214 staining displayed the typical punctate pattern of nucleoporins at the nuclear rim (Figure 2A).

The FG domains of nucleoporins are, due to their amino acid composition, intrinsically disordered and exhibit variable degrees of cohesiveness, which is important for the maintenance of the NPC permeability barrier [52–54]. 1,6-hexanediol (HD) is a mild alcohol that interferes with hydrophobic interactions established between FG repeats [52], thereby disrupting the NPC permeability barrier [55, 56]. To address the potential role of FG repeat cohesion in the formation of NUP214 nuclear bodies, we treated LOUCY cells, which have the most prominent nuclear bodies, with 5% HD and monitored SET-NUP214 nuclear bodies over time by immunofluorescence microscopy. As shown in Figure 2C, untreated cells presented with prominent SET-NUP214 nuclear bodies of different size and frequency. These SET-NUP214 nuclear bodies started to dissolve as early as 2 min after HD treatment and vanished after 10 min HD exposure, as indicated by a largely spread and homogeneous distribution of SET-NUP214 throughout the nucleus (Figure 2C). Hence, we concluded that the formation of SET-NUP214 nuclear bodies depends, at least in part, on cohesive interactions between the FG repeats of NUP214.

### *NUP214* fusions accumulate the nuclear export factor CRM1 in their nuclei and perturb the localization of endogenous nucleoporins

**Figure 3:**
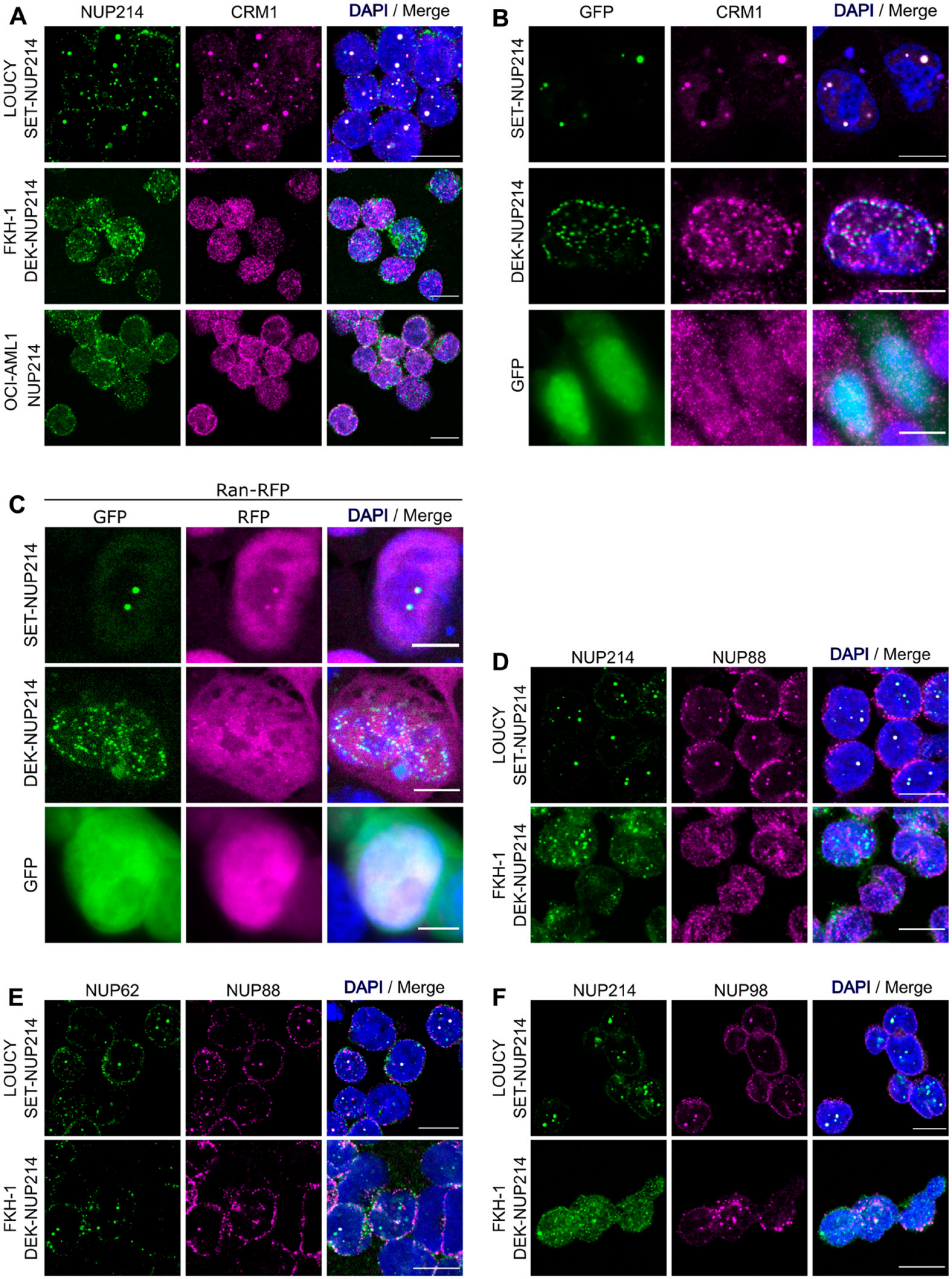
NUP214 fusions sequester the nuclear export factor CRM1 and endogenous nucleoporins to nuclei. (**A**) Cellular distribution of NUP214 (green) and CRM1 (magenta) in leukemia cell lines expressing either SET-NUP214 (LOUCY) or DEK-NUP214 (FKH-1). OCI-AML1 cells were used as control. (**B**) Co-localization of endogenous CRM1 (magenta) with SET-NUP214-GFP and DEK-NUP214-GFP fusion proteins (green) in transiently transfected HCT-116 cells. (**C**) Co-localization of SET-NUP214-GFP and DEK-NUP214-GFP with Ran-RFP in transiently transfected HCT-116 cells. (**D**–**F**) Colocalization of endogenous NUP88 (D), NUP62 (E) and NUP98 (F) with SET-NUP214 in LOUCY and DEK-NUP214 in FKH-1 cells. DNA was visualized with DAPI (blue). Shown are representative epifluorescence images. Scale bars, 10 μm.

We have previously shown that SET-NUP214 in transfected cells interacts with the nuclear export factor CRM1 and that this interaction is enhanced in the presence of RanGTP [22]. Accordingly, we found that SET-NUP214 nuclear bodies accumulate CRM1 in LOUCY (Figure 3A) as well as endogenous CRM1 and exogenous Ran-RFP in transfected HCT-116 cells (Figure 3B and 3C). We made similar observations for endogenous and exogenous DEK-NUP214, however, due to the smaller size of the nuclear foci, the co-localization of the fusion protein with Ran-RFP was less obvious than observed for SET-NUP214 (Figure 3A–3C). In contrast, CRM1 was enriched at the nuclear membrane in OCI-AML1 cells (Figure 3A) and localized throughout the nucleus and cytoplasm in HCT-116 cells expressing GFP alone (Figure 3B), reflecting the normal range of CRM1 distribution in cells (Human Protein Atlas, available from https://www.proteinatlas.org) [57]. To reinforce the notion that NUP214 fusion proteins preferentially bind CRM1-RanGTP nuclear export complexes, we co-expressed SET-NUP214-GFP and DEK-NUP214-GFP in HCT116 cells with RFP-tagged versions of Ran [22]. We employed RanQ69L (RanQ69L-RFP), a non-hydrolyzable mutant of Ran, and RanT24N (RanT24N-RFP), which is resistant to GTP loading and nuclear export complex formation [58]. As expected, both NUP214 fusion proteins sequestered RanQ69L-RFP, but not RanT24N-RFP to nuclear bodies (Supplementary Figure 1A and 1B), complementing our previous biochemical data [22]. Additionally, we observed that the fusion proteins sequestered endogenous NPC components: NUP88 (Figure 3D) and NUP62 (Figure 3E), both of which interact with NUP214 at NPCs, are recruited to SET-NUP214 nuclear foci in LOUCY cells. Similarly, NUP88 and NUP62 co-localized with SET-NUP214-GFP foci in transfected HCT-116 cells (Supplementary Figure 1C and 1D). Interestingly, we also found that NUP98, the main component of the NPC barrier, co-localizes with the nuclear foci of SET-NUP214 (Figure 3E, Supplementary Figure 1E). Similar, albeit to a lesser extent, co-localization of NUP88 (Figure 3D), NUP62 (Figure 3E), and NUP98 (Figure 3F) was observed for DEK-NUP214 in FKH-1 cells as well as in transfected HCT-116 cells (Supplementary Figure 1C and 1D). Altogether, the results suggest that SET-NUP214 and DEK-NUP214 perturb nucleocytoplasmic transport by accumulating CRM1-RanGTP nuclear export complexes and by disturbing the localization of endogenous NPC components.

### *NUP214* fusion proteins are sensitive to nuclear export inhibition

**Figure 4:**
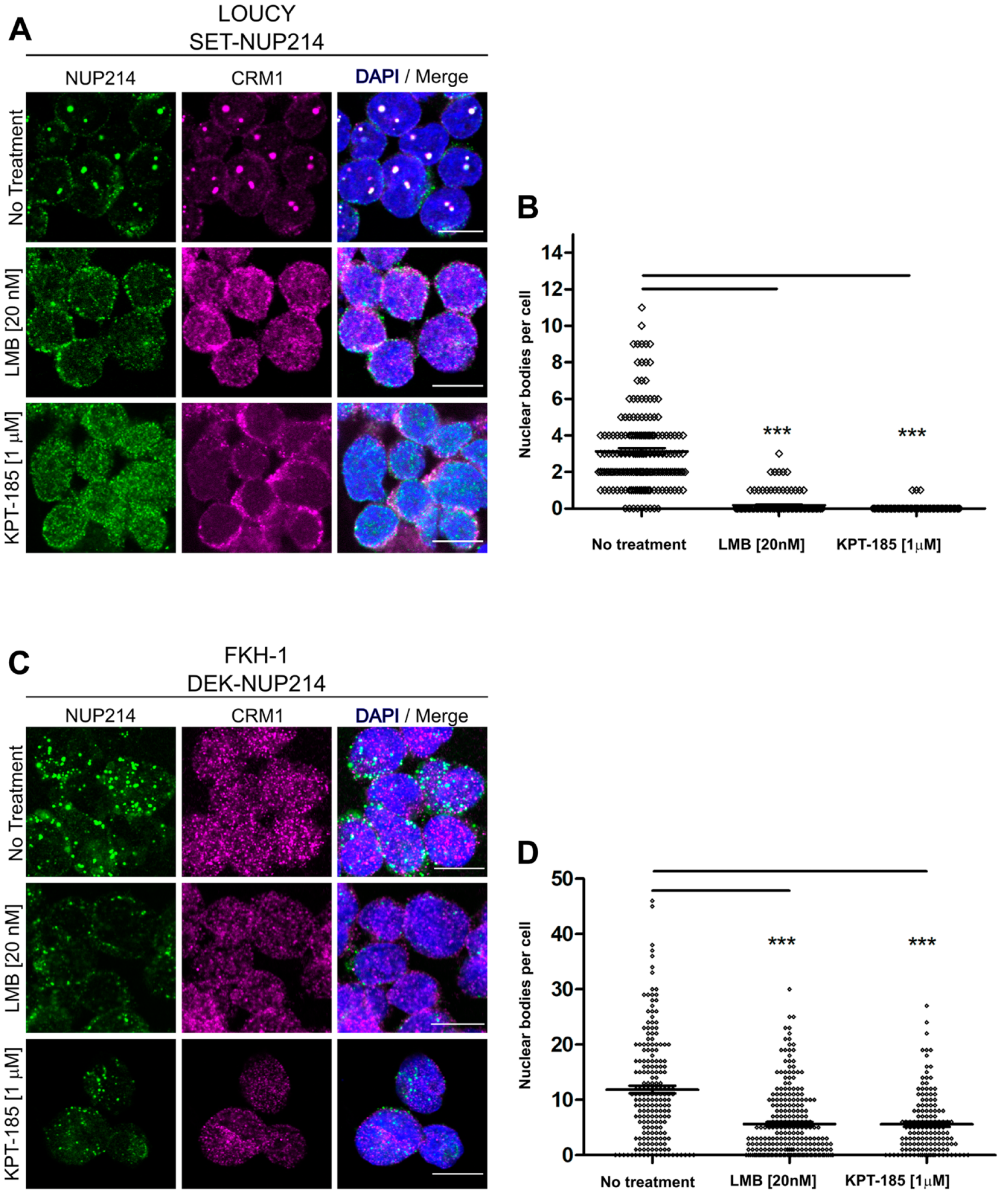
NUP214 fusion proteins are sensitive to inhibition of CRM1. LOUCY and FKH1 cells were treated with (**A**) LMB (20 nM, 3 h) or (**C**) KPT-185 (1 μM, 24 h) and NUP214 (green) and CRM1 (magenta) localization was studied by confocal microscopy. DNA stained with DAPI is depicted in blue. Scale bars, 10 μm. Quantitative analysis of the number of NUP214 nuclear bodies in (**B**) LOUCY and (**D**) FKH-1 cells. At least 150 cells were analyzed for each condition. Statistical differences were calculated using GraphPad Prism (v5.01) using one-way analysis of variance (ANOVA) test. No treatment *vs* LMB; No treatment *vs* KPT-185. ^***^ *p* < 0.001.

CRM1 inhibition by LMB leads to the dissolution of nuclear bodies containing NUP214 fusion proteins, in LOUCY and in transfected cells [22, 23]. We confirmed these previous results for SET-NUP214 in LOUCY cells here (Figure 4A and 4B, Supplementary Figure 1): treatment with LMB (20 nM, 3 h) resulted in a marked dispersal of SET-NUP214 nuclear bodies in LOUCY cells and a localization of SET-NUP214 and CRM1 throughout the nucleus (Figure 4A and 4B, Supplementary Figure 2A). Similar results were obtained after the application of the SINE compound KPT-185 (1 μM, 24 h; Figure 3A and 3B, Supplementary Figure 2A). Treatment with either LMB or KPT-185 led also to a significant decrease in DEK-NUP214 nuclear bodies in FKH-1 cells (Figure 4C and 4D, Supplementary Figure 2A). In transfected cells, the application of LMB and KPT-185 similarly caused the dissolution of SET-NUP214-GFP and DEK-NUP214-GFP nuclear bodies and their homogenous distribution throughout the nucleoplasm, supporting the idea of an interplay between CRM1-mediated nuclear export and leukemia-related NUP214 fusion proteins (Supplementary Figure 2A). The detailed protocol used for quantification of nuclear bodies is presented in Supplementary Figure 2B.

**Figure 5:**
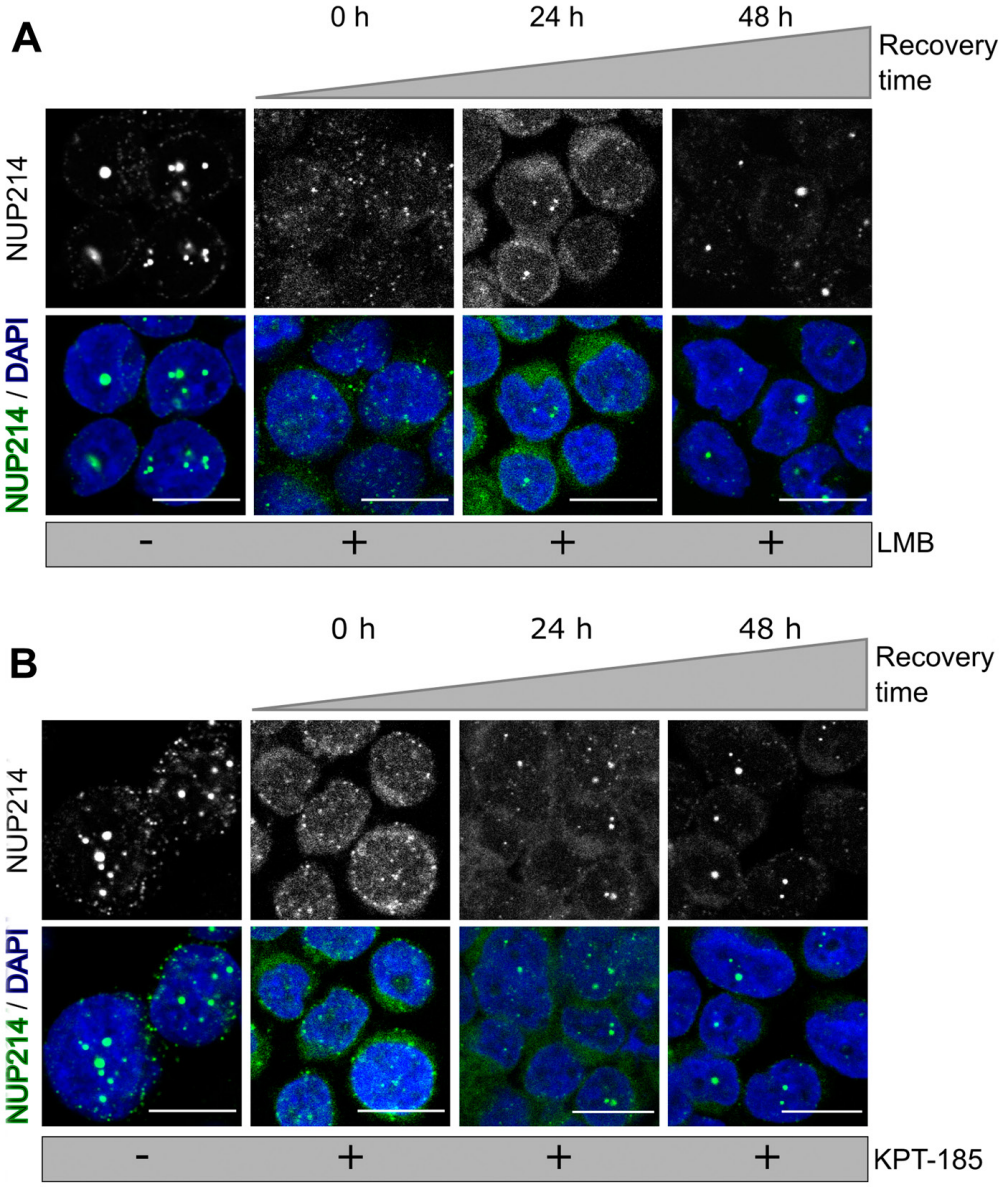
NUP214 nuclear foci reform after withdrawal of CRM1 inhibition. LOUCY cells (SET-NUP214) were treated with (**A**) LMB (20 nM, 3 h) or (**B**) KPT-185 (1 μM, 24 h). Cells were allowed to grow in drug-free medium for up to 48 h, after clearance from the respective drug. The presence of SET- NUP214 nuclear bodies was evaluated by immunofluorescence using anti-NUP214 (green) and anti-CRM1 (magenta) antibodies. DNA stained with DAPI is depicted in blue. Scale bars, 10 μm.

**Figure 6:**
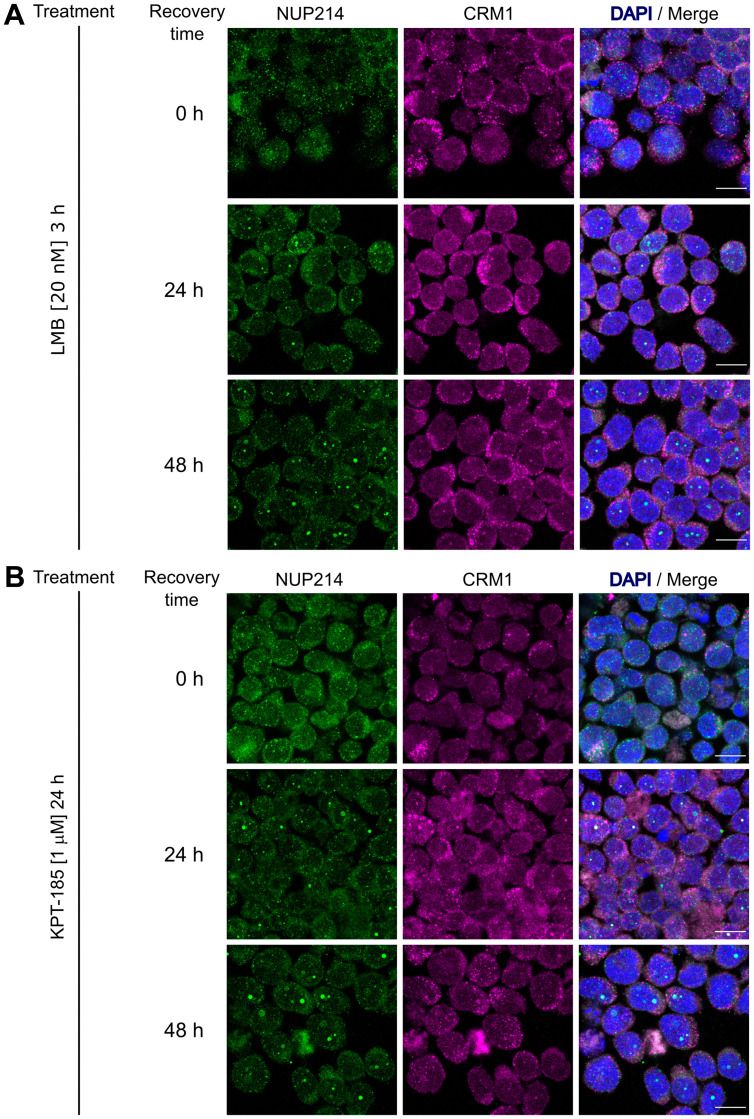
Formation of NUP214 nuclear after withdrawal of CRM1 inhibition is accompanied by CRM1 accumulation. LOUCY cells (SET-NUP214) were treated with (**A**) LMB (20 nM, 3 h) or (**B**) KPT-185 (1 μM, 24 h) and cells were allowed to grow in drug-free medium for up to 48 h after treatment. The formation of SET-NUP214 (green) nuclear foci is accompanied by the accumulation of CRM1 (magenta) in these structures. Shown are representative confocal images. DNA stained with DAPI is depicted in blue. Scale bars, 10 μm.

LMB binds irreversibly to CRM1, while KPT-185 interacts with CRM1 in a slowly reversible fashion [43]. We next examined the effect of drug withdrawal on nuclear body formation of SET-NUP214 in LOUCY cells. No prominent nuclear bodies were detectable immediately after the respective removal of LMB (Figure 5A, 0 h) and KPT-185 (Figure 5B, 0 h). Smaller SET-NUP214 nuclear bodies reformed 24 h after clearance from the drugs (Figure 5A and 5B, 24 h), which were increasing to a similar size as those in non-treated cells within 48 h (Figure 5A and 5B, 48 h and [-]). The re-appearance of SET-NUP214 nuclear bodies was accompanied by accumulation of CRM1 in these structures (Figure 6A and 6B, 24 h and 48 h), supporting the hypothesis that SET-NUP214 localization and nuclear body formation depends on active CRM1.

### Inhibition of CRM1-mediated nuclear export reduces cell viability

**Figure 7:**
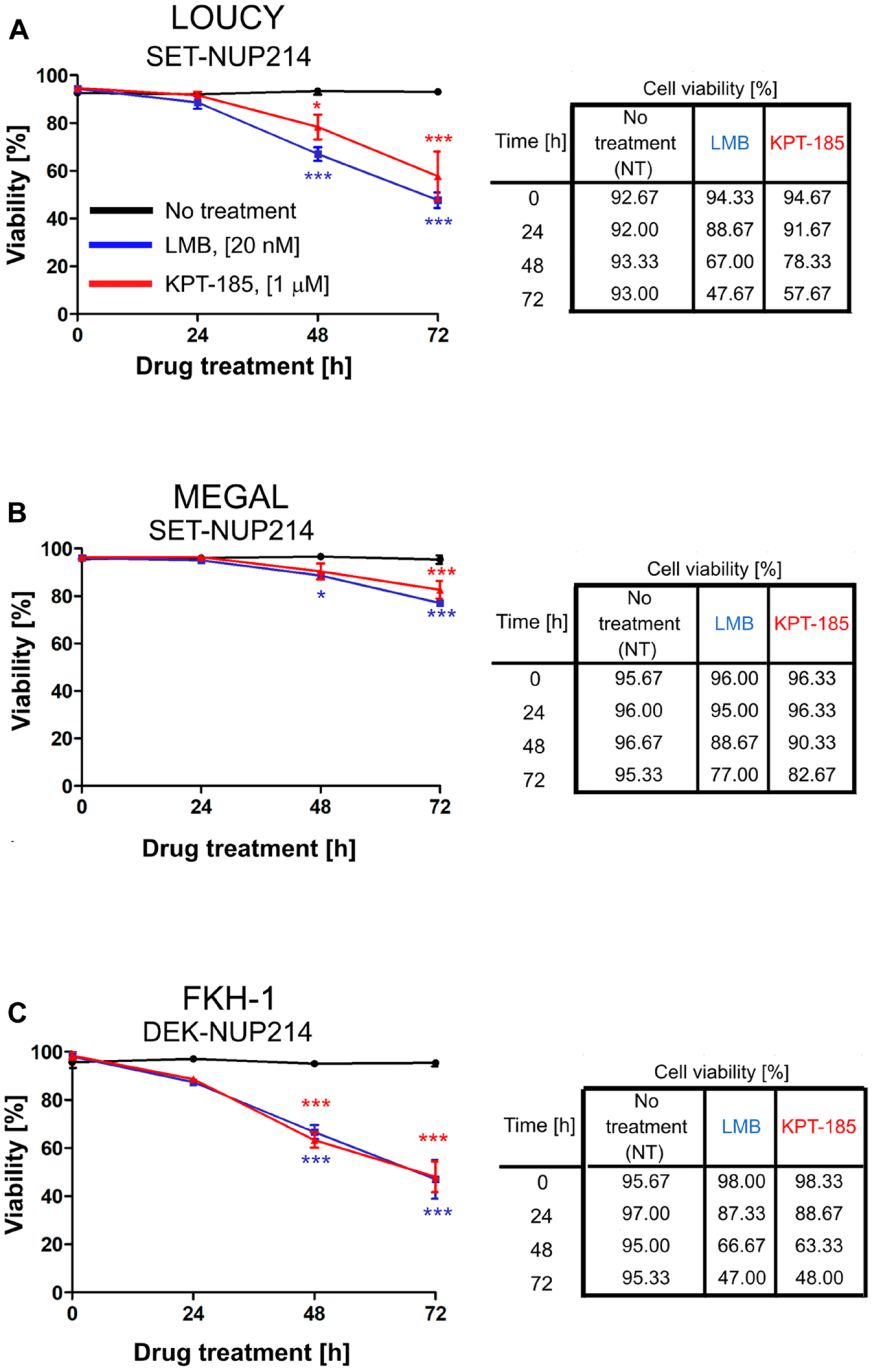
Inhibition of CRM1-mediated nuclear export reduces cell viability. Leukemia cell lines LOUCY (**A**), MEGAL (**B**), and FKH-1 (**C**) were treated with LMB (20 nM) or KPT-185 (1 μM) for the indicated time points and cell viability was measured by Trypan Blue exclusion dye. Statistical differences were calculated using GraphPad Prism (v5.01) using one-way analysis of variance (ANOVA) test. No treatment *vs* LMB; No treatment *vs* KPT-185. ^*^ *p* < 0.05; ^**^ *p* < 0.01; ^***^ *p* < 0.001.

Next, we questioned whether CRM1 inhibition by LMB and KPT-185 affects the survival of leukemia cell lines harboring *NUP214* rearrangements. To address this question, LOUCY, MEGAL, and FKH-1 cells were treated with LMB or KPT-185 up to 72 h and cell viability was assessed at different time-points by Trypan blue exclusion dye assay. As shown in Figure 7A–7C, the viability of LMB- and KPT-185-treated LOUCY, MEGAL, and FKH-1 cells was significantly reduced as compared to untreated cells. Only about 47% of LOUCY (Figure 7A) and FKH-1 (Figure 7C) cells were alive 72 h after exposure to LMB, as compared to 77% of MEGAL cells (Figure 7B). The effect of KPT-185 on cell viability was slightly weaker in LOUCY (Figure 7A) and MEGAL (Figure 7B) cells as compared to LMB, but similar in FKH-1 cells (Figure 7C). Together these data suggest that CRM1 inhibition affects the viability of cells expressing NUP214 fusion proteins and that MEGAL cells appear to be more resistant to CRM1 inhibition in comparison to LOUCY and FKH-1 cells.

### LMB and KPT-185 persistently affect cellular function

**Figure 8:**
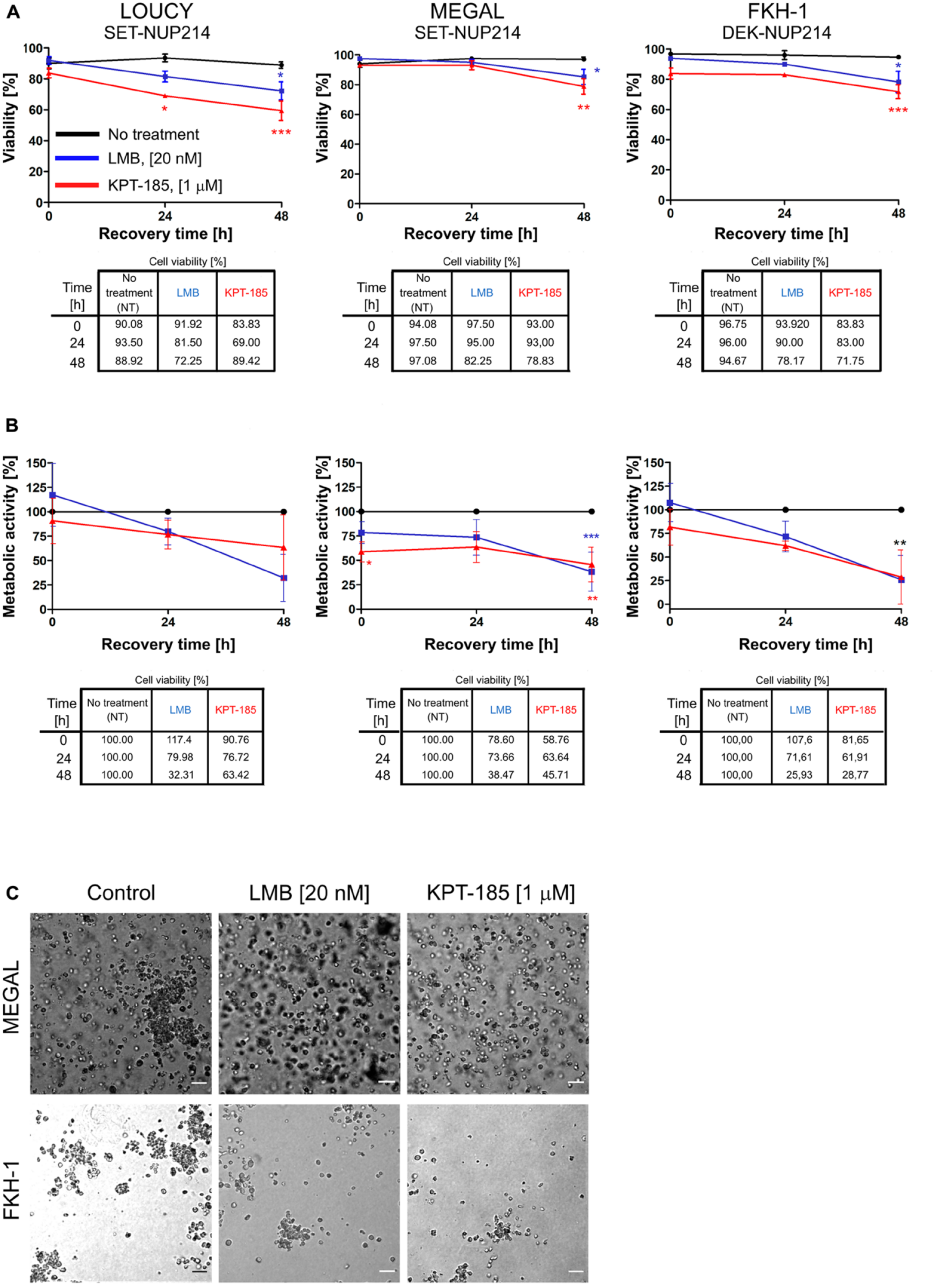
LMB and KPT-185 persistently affect cellular function. LMB or KPT-185 treated LOUCY, MEGAL, and FKH-1 cells were allowed to grow in drug-free medium for up to 48 h after drug clearance. (**A**) Cell viability was determined by Trypan Blue exclusion dye. (**B**) WST-1 assay was performed to measure cellular metabolic activity. Absorbance was measured at 450 nm. Results are normalized to untreated cells and expressed as percentage. (**C**) Colony forming assays of LMB and KPT-185 treated MEGAL and FKH-1 cells. Colonies were visualized under a 10× microscope objective after growth in drug-free Methocult™ medium for 14 days. Scale bars, 100 μm. Statistical differences were calculated using GraphPad Prism (v5.01) using one-way analysis of variance (ANOVA) test. No treatment *vs* LMB; No treatment *vs* KPT-185. ^*^ *p* < 0.05; ^**^ *p* < 0.01; ^***^ *p* < 0.001.

As mentioned above, LMB binds CRM1 irreversibly, in contrast to KPT-185 [43]. This led us to ask how cells respond to treatment withdrawal, i. e. how persistent are the effects of the two CRM1 inhibitors in NUP214-rearranged leukemia cells. We hence exposed the three leukemia cell lines to LMB or KPT-185 as described above and monitored cell viability and metabolic activity at different time points after drug removal. We found that after LMB and KPT-185 removal, cell viability (Figure 8A) and metabolic activity (Figure 8B) of MEGAL and FKH-1 cells were significantly reduced for at least 48 h. Similarly, LOUCY cells showed a significant reduction of cell viability for at least 48 h after drug removal, accompanied by a decrease in their metabolic activity, which nevertheless was not statistically significant. Again, the effect of the two CRM1 inhibitors on cell viability was weaker in MEGAL cells than as in LOUCY and FKH-1 cells (Figure 8A; see also Figure 7). The persistent negative effect of CRM1 inhibition on cellular fitness of the leukemia cell lines was similarly observed in colony-forming assays (see Material and Methods) conducted with MEGAL and FKH-1 cells (Figure 8C). MEGAL cells formed colonies, which were smaller (< 50 μm) than colonies formed by non-treated cells (Figure 8C, top panel), whereas colonies formed by FKH-1 cells were reduced in size and number compared to non-treated cells (Figure 8C, bottom panel). These data further support the notion that MEGAL cells are slightly more resistant to CRM1 inhibition, when compared to LOUCY and FKH-1 cell lines. We additionally performed the experiment with LOUCY cells, however due to the small size of the colonies it was not possible to properly evaluate potential differences between treated and non-treated cells.

### CRM1 inhibition has varying effects on cell proliferation

**Figure 9:**
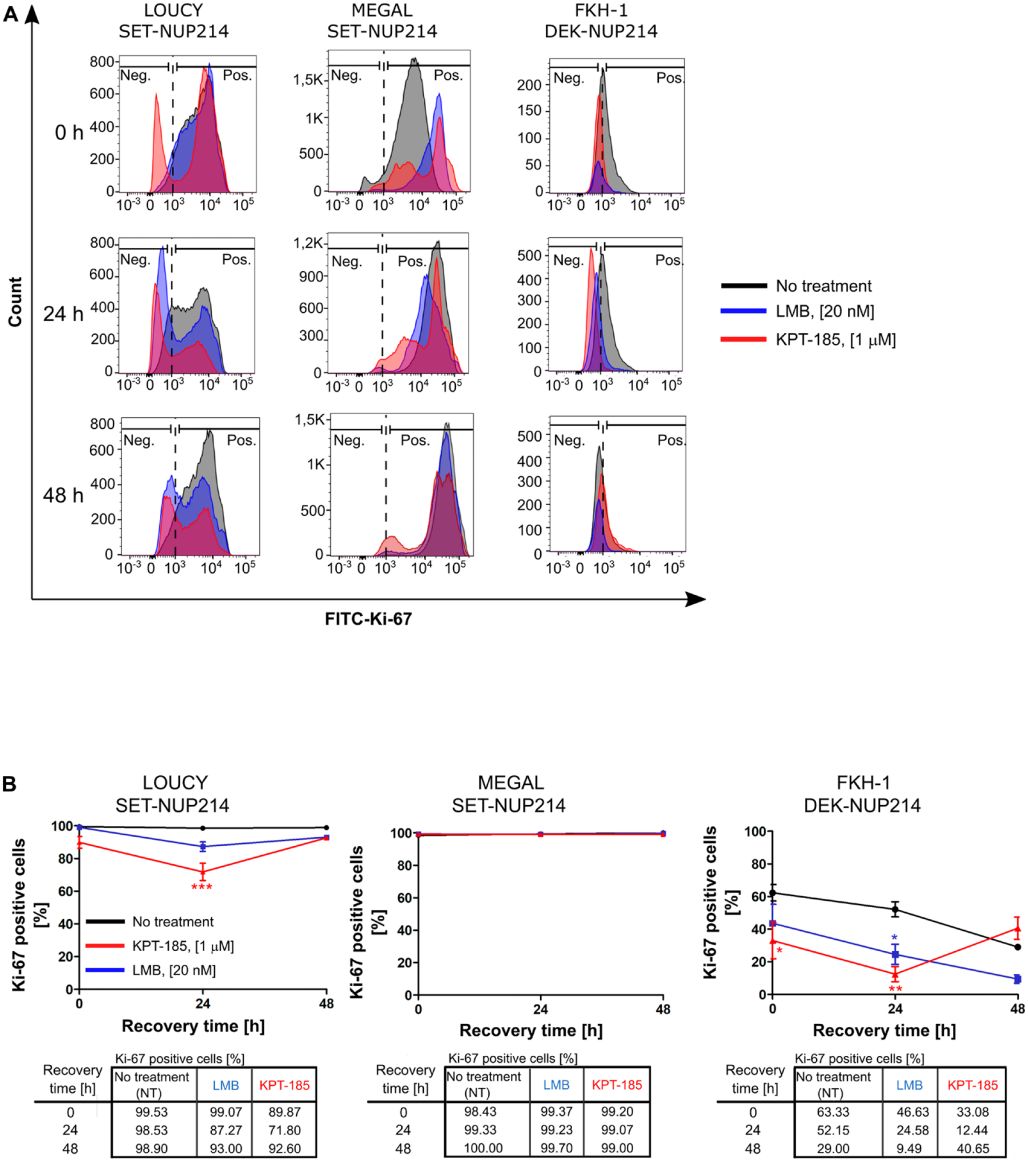
Distinct effects of CRM1 inhibition results on cell proliferation. Flowcytometric analysis of LOUCY, MEGAL, and FKH-1 cells after withdrawal from LMB or KPT-185 treatment. Cell proliferation was evaluated by using FITC-Ki-67 antibodies. (**A**) Histogram representation and (**B**) quantification of FITC-Ki-67 positive cells in the population of single cells at the indicated time points.

We next examined the potential effect of CRM1 inhibitors on cell proliferation: enhanced proliferation is a hallmark of cancer cells. For that, we performed flow cytometry analysis of LOUCY, MEGAL, and FKH-1 cells exposed to LMB or KPT-185 and determined the number of proliferating cells based on the proliferation marker Ki-67 (Ki-67^+^ cells). In LOUCY cells, CRM1 inhibition by LMB had no significant impact on cell proliferation, with a maximum reduction of ~11%, 24 h after the treatment (Figure 9A and 9B, left columns). Moreover, KPT-185 treatment had a significant (*p* < 0.001) effect on cell proliferation, with a 27% reduction of Ki-67^+^ cells, 24 h after the treatment (Figure 9A and 9B, left columns). Proliferation of MEGAL cells was not affected by LMB or KPT-185 treatment (Figure 9A and 9B, middle columns), whereas FKH-1 cell proliferation was significantly lower upon LMB (*p* < 0.05) and KPT-185 (*p* < 0.01) treatment. Proliferation of LOUCY and FKH-1 cells was restored 48 h after KPT-185 removal. Proliferation of LMB-treated FKH-1 cells further decreased 48 h after drug removal, but also non-treated cells were less proliferative.

## DISCUSSION

The pathologic potential of NUP214 fusion proteins has been widely recognized, but the underlying molecular mechanisms have been only sparsely studied [7, 22, 24, 59, 60]. NUP214 is an important player in nuclear export mediated by the β-karyopherin CRM1 and CRM1 inhibition in turn has been proven beneficial in clinical trials as anti-cancer strategy. Here we confirm that endogenous and exogenous SET-NUP214 and DEK-NUP214 form nuclear bodies that accumulate CRM1 and its co-factor RanGTP (Figures 2A and 2C, 3A–3C, Supplementary Figure 1A and 1B). Moreover, both NUP214 chimeras interfere with the cellular distribution of endogenous nucleoporins (Figure 3D–3F, Supplementary Figure 1C–1E), which might have a broad effect on nucleocytoplasmic trafficking. Further in-depth studies would be required to address this possibility.

In agreement with the idea of an interplay between NUP214 fusion proteins and CRM1-mediated nuclear export, two well-known CRM1 inhibitors, leptomycin B (LMB) and the selective nuclear export inhibitor (SINE) KPT-185, led to the dissolution of the SET-NUP214 and DEK-NUP214 nuclear bodies and their homogenous distribution throughout the nucleoplasm (Figure 4A–4D, Supplementary Figure 2). It remains to be established whether NUP214-fusion proteins sequester and trap CRM1 nuclear export complexes, or if CRM1 functions as a scaffold for the assembly of the fusion proteins into nuclear bodies. In any case, the disruption of these structures upon CRM1 inhibition indicates that NUP214 fusion proteins require CRM1 activity for nuclear body formation and possibly for their oncogenic properties. Consistently, treatment of NUP214-rearranged leukemia cell lines with LMB or KPT-185 affects viability, metabolism, and proliferation, albeit to a different extent (Figures 7–9). This effect is sustained even after drug removal (Figure 8), likely due to the stability in drug binding to CRM1. Binding of LMB and KPT-185 to CRM1 is energetically favored, i. e. both drugs not only bind free CRM1, but they also disrupt existing interactions between CRM1 and its cargoes [43]. Binding of LMB to CRM1 results in a covalent, irreversible interaction, whereas multiple hydrophobic bridges between KPT-185 and CRM1 result in a slowly reversible interaction [40, 43, 61].

MEGAL cells respond different to CRM1 inhibition compared to LOUCY (and FKH-1) cells, i. e. they appear to be more robust/resistant (Figures 7–9). These discrepancies between MEGAL and LOUCY cells, which both express SET-NUP214, may be explained by differences in CRM1 levels in these two cell lines. Indeed, preliminary data indicate that in particular KPT-185 treatment coincided with reduced CRM1 levels in LOUCY cells and to a lesser extent in MEGAL cells and was also sustained after drug removal although some recovery could be observed (Supplementary Figure 3A and 3B). LMB treatment on the contrary appears to not alter CRM1 protein levels. Further detailed analyses are required here, but our results are in accordance with previous reports which have shown that KPT-185 treatment led to reduced CRM1 protein levels, but not so LMB [26, 31, 34, 62, 63]. Cellular response to CRM1 inhibitors in leukemia has moreover been reported to depend on the mutational status of the tumor suppressor *TP53* [27]. LOUCY cells harbor a missense mutation p53^V272M^ (Supplementary Figure 4A), which results in functionally inactive p53 [65, 66]. MEGAL cells feature a heterozygous frameshift mutation in *TP53* due to deletion of cytosine at position 898, resulting in a frameshift after a leucine residue at position 299, which affects p53’s 42 C-terminal residues (c. del898C, p53^299fs*42^; Supplementary Figure 4A). This mutant form of p53 in MEGAL cells is comparatively more abundant than the wild-type protein (Supplementary Figure 4B). The biological implications of p53^299fs*42^ remain to be elucidated. However, one may hypothesize that p53^299fs*42^ acts as dominant negative and hinders the biological function of the wild type counterpart, which may be stabilized by CRM1 inhibition [67, 68]. Such a dominant-negative effect of p53^299fs*42^ may contribute to the reduced sensitivity of MEGAL cells to CRM1 inhibition. Such a reduced sensitivity may arise from a reduced nuclear accumulation of tumor suppressor genes, delayed cell cycle, and/or reduced variation in overall protein expression after CRM1 inhibition, as seen in a fibrosarcoma cell line treated with the SINE KPT-330 [69]. However, future studies are required to further elucidate this possibility. Moreover, the distinct genetic backgrounds of the cell lines may contribute to their respective response to CRM1 inhibition (Supplementary Table 1). Although LOUCY and MEGAL cells both carry del (9)(q34.11q34.13) that originates the *SET-NUP214* fusion transcript, the two cell lines are etiologically distinct and exhibit profound karyotypic differences (Supplementary Table 1). Whereas LOUCY cells were isolated from a patient with T-ALL (French-American-British, FAB, classification FAB-L2) and exhibit a hypodiploid karyotype, MEGAL cells derive from an AML (FAB-M7) patient and carry a hyperdiploid karyotype (Supplementary Table 1) [64].

Taken together, our results suggest that CRM1 inhibition might be an interesting therapeutic option in NUP214-related leukemia, similarly as described for several cancer models, including other forms of leukemia [29, 30, 33, 47, 70]. This notion is further supported by a recent study that showed successful disease remission for an AML patient with NUP214 driven leukemia who was treated with KPT-330 as single agent [71, 72]. This is particularly important, since no specific or targeted therapy has been developed so far [71].

## MATERIALS AND METHODS

### Cell lines

LOUCY (T-cell leukemia), MEGAL (acute megakaryoblastic leukemia), FKH-1 (acute myelocytic leukemia), OCI-AML1 (acute myeloid leukemia), and MOLM-13 (acute myeloid leukemia) cell lines were purchased from the Leibniz Institute - DSMZ-German Collection of Microorganisms and Cell Cultures GmbH (Braunschweig, Germany). For details see Supplementary Table 1. HCT-116 cells were a gift from Dr. Denis Lafontaine (Institute of Molecular Biology and Medicine, Université Libre de Bruxelles, Gosselies, Belgium). Cell lines were maintained as detailed in the Online Supplementary Methods.

### Plasmids and transfections

To generate SET-NUP214-GFP, total RNA was extracted from LOUCY cells using the High Pure RNA Isolation Kit (Roche Life Sciences, Basel, Switzerland) according to the manufacturer’s instructions. cDNA synthesis was performed by reverse transcription-PCR. Cloning of SET-NUP214 is described in the Online Supplementary Methods.

The pENTR1-DEK-NUP214 plasmid was a gift from Dr. Martin Ruthardt (Cardiff University, UK) and was subcloned into the peZY-EGFP destination vector using the Gateway™ LR clonase™ enzyme mix (Invitrogen, Merelbeke, Belgium), as described in the Online Supplementary Methods.

### Immunofluorescence of suspension cells

Leukemia cells were seeded at 0.8 × 10^6^ cells/ml and grown for 24 h - 48 h. Cells were fixed and processed for immunofluorescence as detailed in the Online Supplementary Methods.

### Immunofluorescence of adherent cells

HCT-116 cells were seeded on polylysine-coated glass coverslips 24 h prior to transfection. After transfection, cells were fixed in 2% formaldehyde and processed for immunofluorescence as detailed in the Online Supplementary Methods.

### 1,6-hexanediol treatment

LOUCY cells were seeded at 0.8 × 10^6^ cells/ml 24 h prior to treatment. Cells were then incubated with 5% 1,6-hexanediol (Sigma-Aldrich, Overijse, Belgium) for different time-points. After treatment, cells were washed and processed for immunofluorescence as detailed in the Online Supplementary Methods.

### Inhibition of CRM1-mediated nuclear export

Cells were seeded at 0.8 × 10^6^ cells/ml for 24 h. Next, cells were treated with 1 μM KPT-185 (Selleck Chemicals, Munich, Germany) or 20 nM LMB (Enzo Life Sciences, Brussels, Belgium). Cell viability and proliferation were assessed by (i) Trypan Blue exclusion dye assay, (ii) the cell proliferation reagent WST-1 (Roche Life Sciences) according to the manufacturer’s instructions, and (iii) flow cytometry using FITC-Ki-67 (BD Biosciences, CA, USA). The detailed protocols are described in the Online Supplementary Methods.

### Statistics

Experiments were performed at least three times and the results represent the mean ± SEM for three independent biological replicates. Plots were generated and statistical analysis was performed using GraphPad Prism (Version 5.01; GraphPad Software Inc., CA, USA). Statistical differences were calculated by one-way analysis of variance (ANOVA). During evaluation of the results a confidence interval α of 95% and *p* values lower than 0.05 were considered as statistically significant. Significance levels are represented as ^*^ *p* < 0.05, ^**^ *p* < 0.01 or ^***^ *p* < 0.001.

## SUPPLEMENTARY MATERIALS


